# Development and validation of a predictive risk model based on retinal geometry for an early assessment of diabetic retinopathy

**DOI:** 10.3389/fendo.2022.1033611

**Published:** 2022-11-21

**Authors:** Minglan Wang, Xiyuan Zhou, Dan Ning Liu, Jieru Chen, Zheng Zheng, Saiguang Ling

**Affiliations:** ^1^ Department of Ophthalmology, Second Affiliated Hospital of Chongqing Medical University, Chongqing, China; ^2^ School of Computer Science and Engineering, Faculty of Engineering, University of New South Wales, Sydney, NSW, Australia; ^3^ Institute of EVision Computing, EVision technology (Beijing) co. LTD, Beijing, China

**Keywords:** nomogram, prediction model, retinal geometry, diabetic retinopathy, T2DM

## Abstract

**Aims:**

This study aimed to develop and validate a risk nomogram prediction model based on the retinal geometry of diabetic retinopathy (DR) in patients with type 2 diabetes mellitus (T2DM) and to investigate its clinical application value.

**Methods:**

In this study, we collected the clinical data of 410 patients with T2DM in the Second Affiliated Hospital of Chongqing Medical University between October 2020 and March 2022. Firstly, the patients were randomly divided into a development cohort and a validation cohort in a ratio of 7:3. Then, the modeling factors were selected using the least absolute shrinkage and selection operator (LASSO). Subsequently, a nomogram prediction model was built with these identified risk factors. Two other models were constructed with only retinal vascular traits or only clinical traits to confirm the performance advantage of this nomogram model. Finally, the model performances were assessed using the area under the receiver operating characteristic curve (AUC), calibration plot, and decision curve analysis (DCA).

**Results:**

Five predictive variables for DR among patients with T2DM were selected by LASSO regression from 33 variables, including fractal dimension, arterial tortuosity, venular caliber, duration of diabetes mellitus (DM), and insulin dosage (P< 0.05). A predictive nomogram model based on these selected clinical and retinal vascular factors presented good discrimination with an AUC of 0.909 in the training cohort and 0.876 in the validation cohort. By comparing the models, the retinal vascular parameters were proven to have a predictive value and could improve diagnostic sensitivity and specificity when combined with clinical characteristics. The calibration curve displayed high consistency between predicted and actual probability in both training and validation cohorts. The DCA demonstrated that this nomogram model led to net benefits in a wide range of threshold probability and could be adapted for clinical decision-making.

**Conclusion:**

This study presented a predictive nomogram that might facilitate the risk stratification and early detection of DR among patients with T2DM.

## Introduction

It is estimated that more than 600 million individuals will be reported with diabetes mellitus (DM) worldwide by 2040 (1); 85% of these individuals will have type 2 diabetes mellitus (T2DM) (2), and more than one-fifth will suffer from diabetic retinopathy (DR) (3). DR is one of the vascular-incompetent complications of DM, resulting from chronic hyperglycemia. It is the leading cause of preventable blindness in the working population (4). This severe sight-threatening complication is characterized by irreversible visual loss and progressive retinal circulation damage, manifested as patches of retinal non-perfusion and neovascularization, pathologically (5, 6).

Although the pathogenesis of DR is not completely clear to date, several risk factors have been confirmed, including increased hemoglobin A1c (HbA1c) level, chronic hyperglycemia, and duration of DM (4, 7). However, these traditional risk factors cannot explain the risk variation of DR completely (8). Identifying additional risk factors beyond these conventional and recognized factors is of significant importance to the early detection and better management of DR, especially the risk factors related to retinal vascular incompetence.

In the last decade, the retinal vascular geometry (the retinal vascular morphology) of DR has gained prominence in emerging studies. The retinal vascular tree is the only site lacking autonomic innervation (9), spreading across the whole retina and modifying itself to fit local metabolism. The retinal vascular morphology is the key measure of local perfusion and metabolism status, indirectly reflecting the retinal function. Chronic high blood sugar contributes to abnormal serum, disrupting the balance of self-regulation of retinal vessels and leading to a higher risk of incident DR. Although a mature treatment plan for DR has been applied clinically according to correlative clinical guidelines, predicting the probability of incident DR precisely, screening high-risk individuals efficiently in the early phase, and determining the timing of medical intervention accurately are still issues demanding for solutions at present.

A nomogram is a useful tool to predict the accurate probability of clinical events, especially adverse outcomes, aiding to meet the drive for individualized medicine (10). It can transform a complicated calculation formula into a scoring system (11), intuitively showing the numerical correlation between a specific event and risk factors. This study aimed to develop a predictive nomogram model to screen the risk of DR among patients with T2DM. Early detection and identification of high-risk populations may facilitate early treatment and a better prognosis.

## Methods

### Research participants

We collected and analyzed the data of 410 patients with T2DM in the Second Affiliated Hospital of Chongqing Medical University between October 2020 and March 2022. The inclusion criteria included 1) T2DM with or without DR, diagnosed according to relevant criteria (fasting plasma glucose ≥ 7.0 mmol/L or 2-h plasma glucose ≥ 11.1 mmol/L or HbA1c ≥ 6.5%) (12); 2) no other retinopathy; and 3) complete clinical data. The exclusion criteria included 1) other co-occurring retinopathy-like retinal vascular occlusion, age-related macular degeneration, and so forth; 2) axial length >25 mm; 3) incomplete data; 4) previous interventions (retinal laser photocoagulation, intravitreal injection, and vitrectomy); and 5) blurred digital fundus image.

After the aforementioned screening, a total of 410 participants were recruited in this study and randomly divided into a training cohort (n = 292) and a validation cohort (n = 118) in a ratio of 7:3 using R software. Many variables showed statistically significant differences between DR and non-diabetic retinopathy (NDR), such as hypertension, serum creatinine level, and duration of DM. These nonhomogeneous variables were potential factors for modeling. More details are presented in **Table 1**.

**Table 1:** Characteristics of the patients in the NDR and DR.

Characteristics	NDR (n = 182)		DR (n = 228)		P
	Mean	SD/Percentage	Mean	SD/Percentage	
Age (years)	62.12	0.0968	64.2000	10.7480	0.132
Gender (Female)	93	51.1%	123.0000	53.90%	0.566
Duration (years)	6.058	3.4002	9.1180	4.5801	<0.001*
Oral drug only (yes)	122	67%	90.0000	39.50%	<0.001*
Insulin dose (IU)	9.786	16.3549	20.6080	20.9781	<0.001*
BMI (kg/m^2^)	24.2318	3.312	24.7295	3.7429	0.072
Systolic blood pressure (mmHg)	131.2	16.821	139.2500	19.2050	0.038
Diastolic blood pressure (mmHg)	81.82	13.45	80.5100	13.4190	0.198
Hypertension (yes)	93	51.1%	149.0000	65.40%	0.004
Smoking history (yes)	57	31.3%	57.0000	25.00%	0.156
Mean branch angle (°)	54.2921	0.7497	53.2071	10.1054	0.032
Fractal dimension	1.5584	0.0358	1.5131	0.0531	<0.001*
Arterial tortuosity	0.6039	0.1274	0.7402	0.4000	<0.001*
Venular tortuosity	0.819	0.138	0.8776	0.1991	0.002
Arterial caliber (μm)	48.5279	3.2934	48.4769	4.2410	0.487
Venular caliber (μm)	68.3851	5.2224	73.6248	7.0740	<0.001*
AVR	0.7137	0.5277	0.6629	0.6782	0.002
HbA1c (%)	8.5129	1.8219	8.7895	2.1703	0.419
eGFR (ml/min/1.73 m^2^)	98.402	21.4759	82.9920	28.5300	<0.001*
Creatinine (μmol/L)	9.1145	2.9815	10.3861	5.1095	0.001
Fasting plasma glucose (mmol/L)	9.1145	2.9815	10.3861	5.1095	0.181
A/G	1.5884	0.3739	1.4424	0.3039	<0.001*
Triglyceride (mmol/L)	1.8976	1.1818	2.0131	1.9573	0.084
TC (mmol/L)	4.4555	1.0263	4.5810	1.1400	0.206
HDL (mmol/L)	1.1520	0.3579	1.1328	0.3038	0.946
LDL (mmol/L)	2.3223	0.7051	2.4768	0.8359	0.112
ApoA1 (mmol/L)	1.6296	5.2539	1.2940	0.2700	0.055
ApoB (mmol/L)	0.8775	0.2597	0.8213	0.2510	0.027
ApoE (mmol/L)	46.1534	24.7181	40.8618	17.8197	<0.001*
Lipoprotein (mmol/L)	220.1080	267.0026	255.8300	267.1131	0.043
NEFA (mmol/L)	1.1201	9.5043	0.3629	0.2405	0.004
Urinary albumin-to-creatinine ratio	4.1964	15.3784	50.4970	156.3525	<0.001*
Albuminuria (mmol/L)	31.2920	112.3517	301.3360	1026.3131	<0.001*

AVR, arteriole-to-venule ratio; BMI, body mass index; HbA1c, hemoglobin A1c; HDL, high-density lipoprotein; LDL, low-density lipoprotein; TC, total cholesterol; A/C, albumin-to-globulin ratio; eGFR, estimated glomerular filtration rate; SD, standard deviation.

Continuous variables are presented as mean and SD, and categorical variables are presented as mean and percentage. P-values were compared by independent t-test, Mann–Whitney U test, or chi-square test as appropriate. *P < 0. 001.

### Retinal vascular geometry measurement

The retinal vascular traits were measured with the optic-centered fundus images, and the required digital images were obtained using a high-resolution TOPCON TRC-NW8 digital retinal camera (Topcon Optical Company, Tokyo, Japan) at a 45° angle after pupil dilation. All study participants underwent fundus fluorescein angiography (FFA) to determine their retinal status. These photos were classified as DR and NDR by two experienced professors (XZ and DL) according to the Early Treatment Diabetic Retinopathy Study and the result of the FFA (13).

To ensure the accuracy of the measurements, two researchers measured the retinal vessel characteristics independently (SL and ZZ) using digital fundus image analysis software EVisionAI. This software was based on bionic human vision and deep learning system and could automatically recognize and segment retinal features. If different consequences were obtained from the two experts, another measurement was required. The right fundus image was collected. The left fundus image was an alternative if the poor image led to a blurred view of the retinal vessel.

First, the digital images were uploaded to the software, and the position of the optic disc and fovea was located automatically. Then, the software automatically sketched the map of the retinal geometry. At the same time, the vessels were classified as venule and arteriole. The operator edited the incorrectly labeled vessels. Furthermore, the vascular traits were quantified using a built-in computerized algorithm.

The vascular traits in this study included vascular caliber, tortuosity, fractal dimension, arteriole-to-venule ratio (AVR), and mean branch angle. The arterial diameter was defined as the average diameter of all visible arteries in the fundus image; the venular diameter was also measured similarly. The AVR was defined as the ratio of the arterial diameter to the venous diameter in the same image measured in an aforementioned manner. The vascular tortuosity was measured as the mean value of the tangent slope at every point on the pathway of tortuous vessels. The fractal dimension was measured on the basis of self-similarity, reflecting how the branching pattern filled the space, describing the complexity of the retinal vascular tree effectively. In a word, the more complex the vascular texture, the larger the fractal dimension value was. The mean branch angle is the average measurement of all of the branching angles within the area of two disc diameters away from the optic disc boundary.

### Observation indexes

The clinical data including age, sex, duration of DM, smoking history, HbA1c, fasting plasma glucose, insulin dosage, hypertension, and so forth were collected. The retinal vascular parameters were measured, including mean vascular caliber, AVR, mean vascular tortuosity, fractal dimension, and mean branch angle. Two researchers (MW and XZ) checked the collected data independently to ensure completeness and reliability.

### Statistical analysis

**Figure 1:**
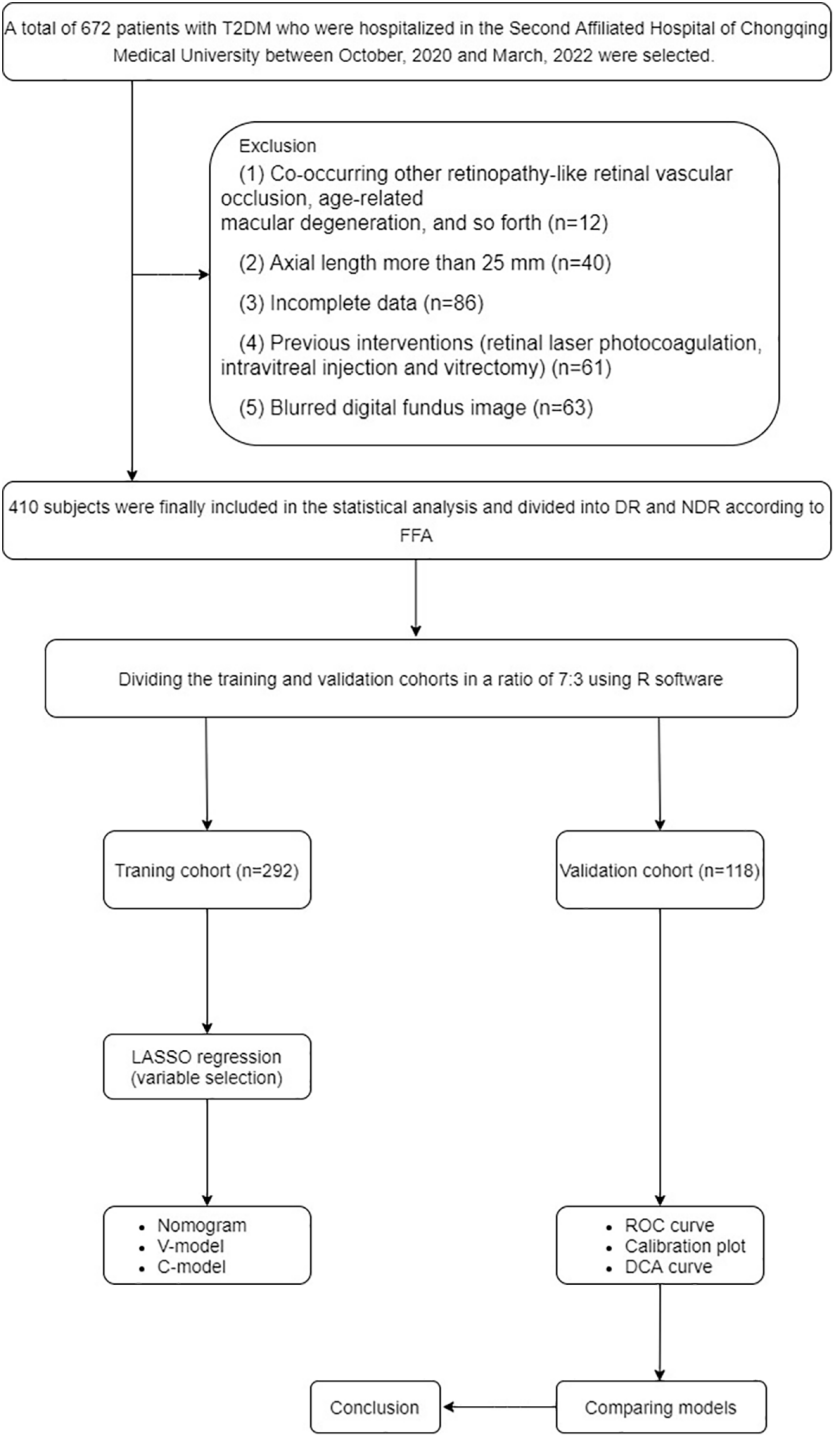
Flow diagram of the analysis.

The statistical analysis was performed on IBM SPSS version 23 software and R software (version 4.2.1). All continuous variables were checked for normality. The categorical variables were displayed as counts and percentages and analyzed using the χ^2^ test. The continuous variables were displayed as means ± standard deviation and analyzed using the t-test or Mann–Whitney U test. The independent predictors for DR among patients with T2DM were identified by the least absolute shrinkage and selection operator (LASSO) regression. Before selection, all of the feature values were standardized using the Z-score to remove dimension divergence among the observational data. The discrimination, calibration, and clinical application value of the prediction models were validated in both training and validation cohorts with areas under the receiver operating characteristic curve (AUCs), calibration plots, and decision curve analysis (DCA) curves. A P value < 0.05 indicated statistically significant differences. The details were presented in **Figure 1**.

## Results

### General characteristics of the participants

A total of 410 patients with T2DM, including 292 in the training cohort and 118 in the validation cohort, participated in this study. In the training cohort, 160 patients developed DR, accounting for 54.79%. In the validation cohort, 68 patients developed DR, accounting for 57.63%. No significant differences were observed in sex, age, and Body Mass Index (BMI) between DR and NDR (P > 0.05), indicating comparability between the two groups. More details are presented in **Table 1**.

### Predictors selection

**Figure 2:**
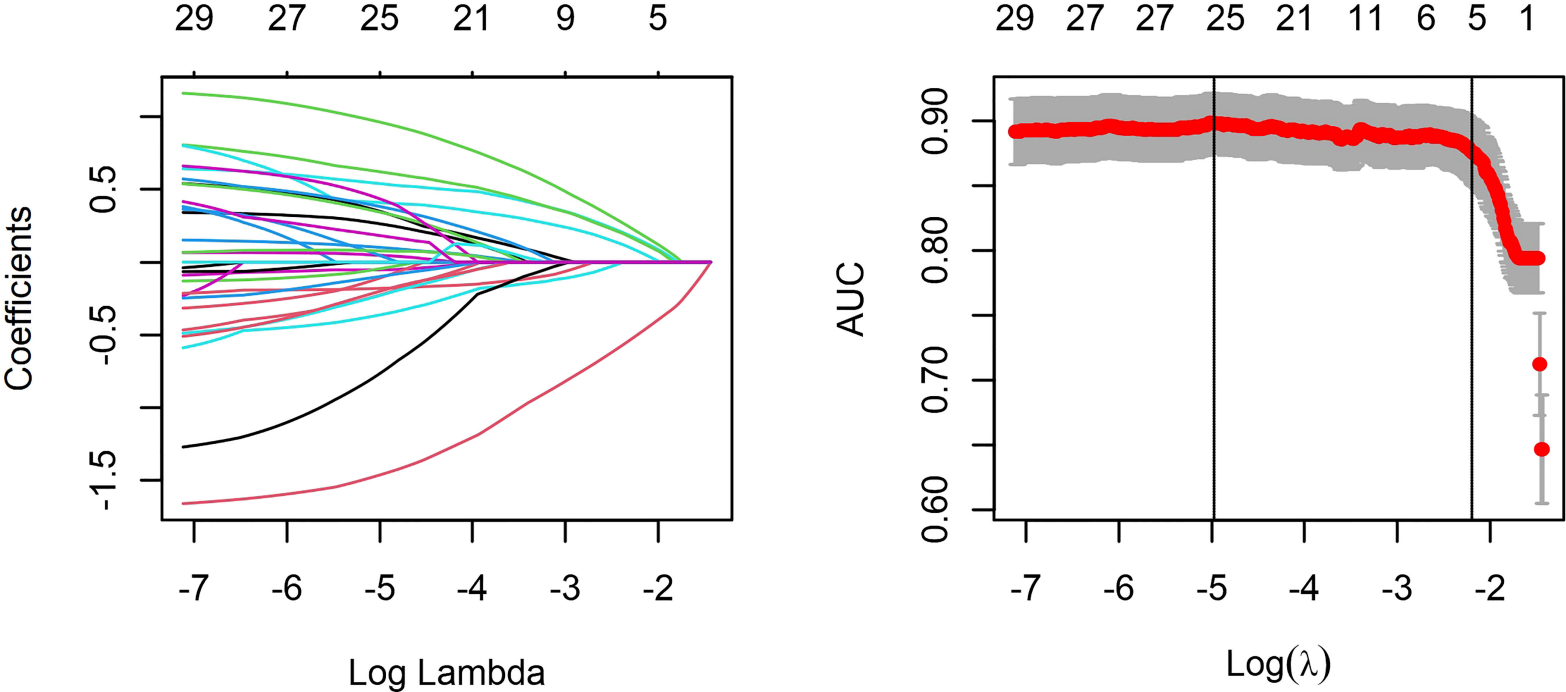
Least absolute shrinkage and selection operator (LASSO) regression and variable selection. All features were normalized by Z-score to remove the dimension divergence among the observational variables. In the cross-validated error plot, 25 variables with non-zero coefficients were selected at minimum cross-validated error, while five variables with non-zero coefficient remained at minimum cross-validation error within 1 standard error of the minimum.

The LASSO regression analysis can select the independent risk factors for the linear regression models by shrinking the regression coefficients of variables toward zero. Supposing the regression coefficient is zero, the corresponding variable is excluded from the model. The remaining factors with non-zero regression coefficients are called independent risk factors having a closer association with the outcome event. In this study, 33 variables were introduced, and five variables with non-zero regression coefficients remained (**Figure 2**). The features selected in the LASSO analysis were fractal dimension, venular caliber, arterial tortuosity, insulin dosage, and duration of T2DM.

### Development of models

**Figure 3:**
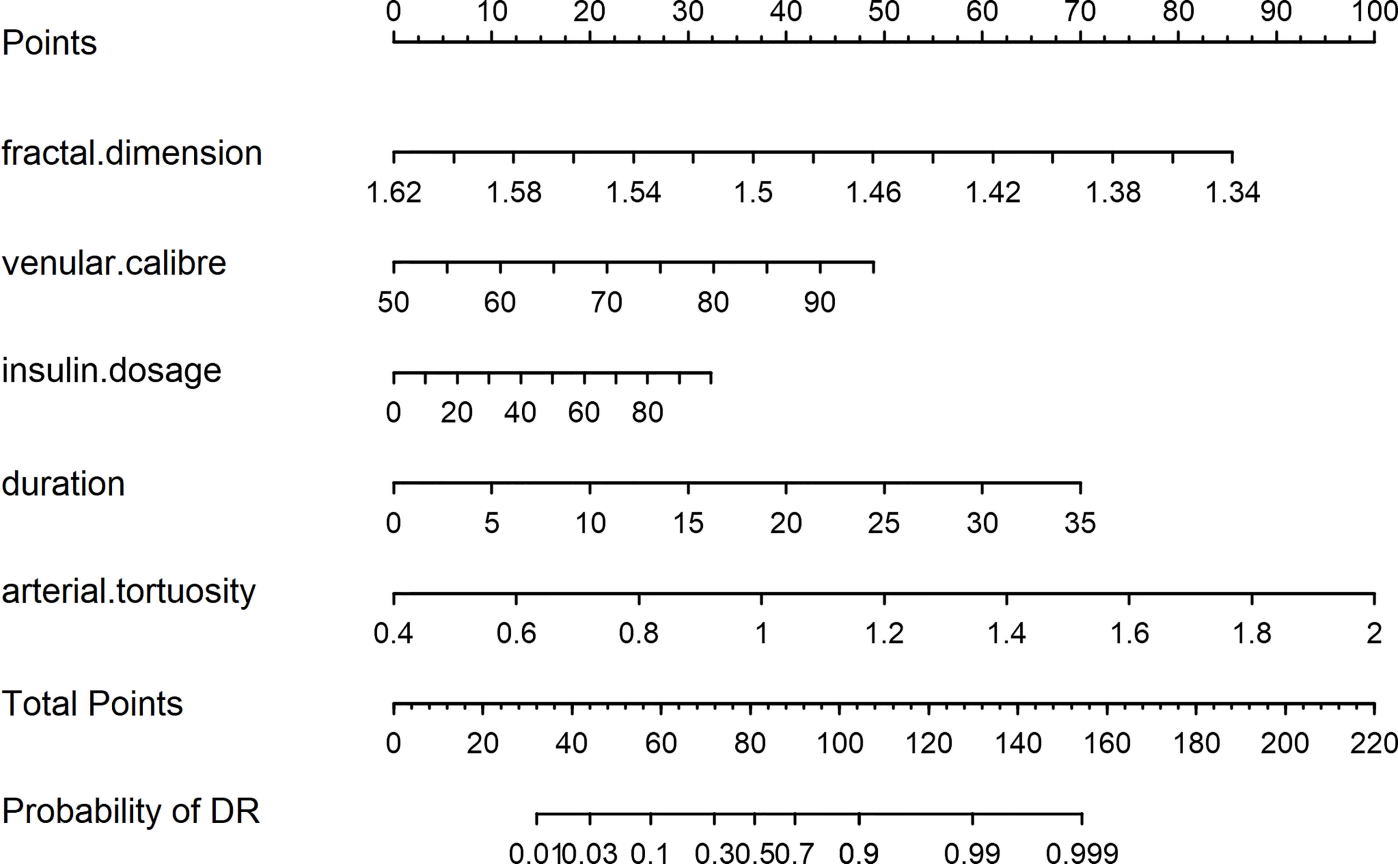
The nomogram model.

A nomogram model was developed for DR risk prediction with these selected features using the multivariable logistic regression analysis (**Figure 3**). The probability of incident DR was calculated using the following equation:


logist(P)=31.4591−28.7245×fractal dimension+0.1022×venular caliber+5.8777×arterial tortuosity+0.0304×insulin dosage+0.1881×duration of T2DM

**Figure 4:**
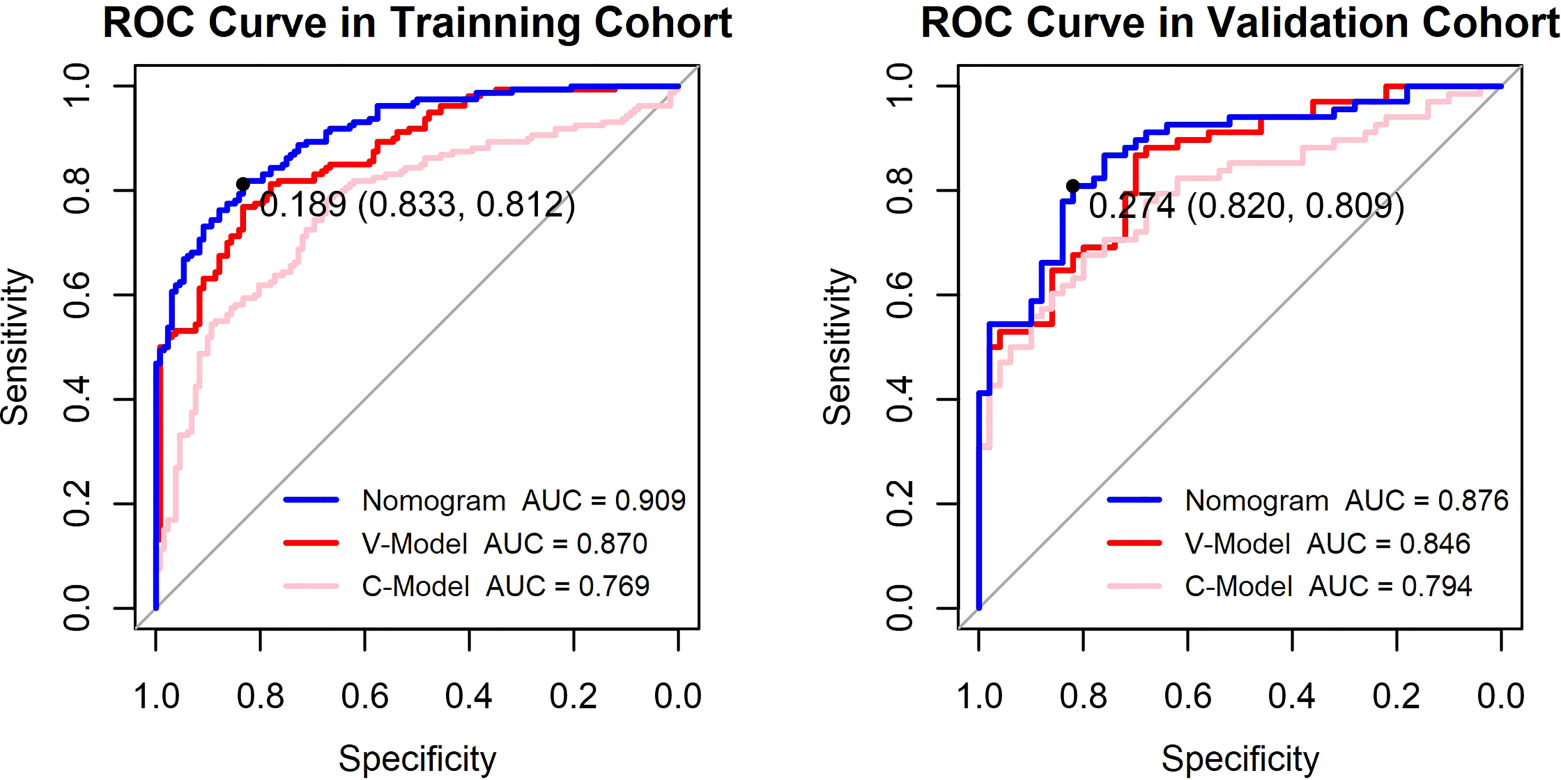
Receiver operating characteristic (ROC) curves of the constructed models. The y-axis means the true-positive rate of the risk prediction. The x-axis means the false-positive rate of the risk prediction. The blue line represents the performance of the nomogram. The cutoff value and area under the receiver operating characteristic curve (AUC) are presented in the picture as well.

Additionally, two models were constructed to confirm the performance advantage of the nomogram. The clinical model (C-Model) was built by a previous study (14) with clinical signatures (age, HbA1c, triglyceride, albuminuria, and duration of DM). The selected retinal vascular traits (fractal dimension, venular caliber, and arterial tortuosity) were used to establish the vascular geometry model (V-Model). According to the receiver operating characteristic (ROC) curves, the performance of the V-Model was better than that of the C-Model in both training and validation cohorts (**Figure 4**). This indicated that the retinal vascular signatures exhibited more predictive value than that of the clinical data. However, the predictive nomogram model displayed the highest AUC among these three (0.909 in the training cohort and 0.876 in the validation cohort). Accordingly, the retinal vascular traits combined with the clinical data of patients could improve the specificity and sensitivity of diagnosis.

## Validation of the models

### Calibration

**Figure 5:**
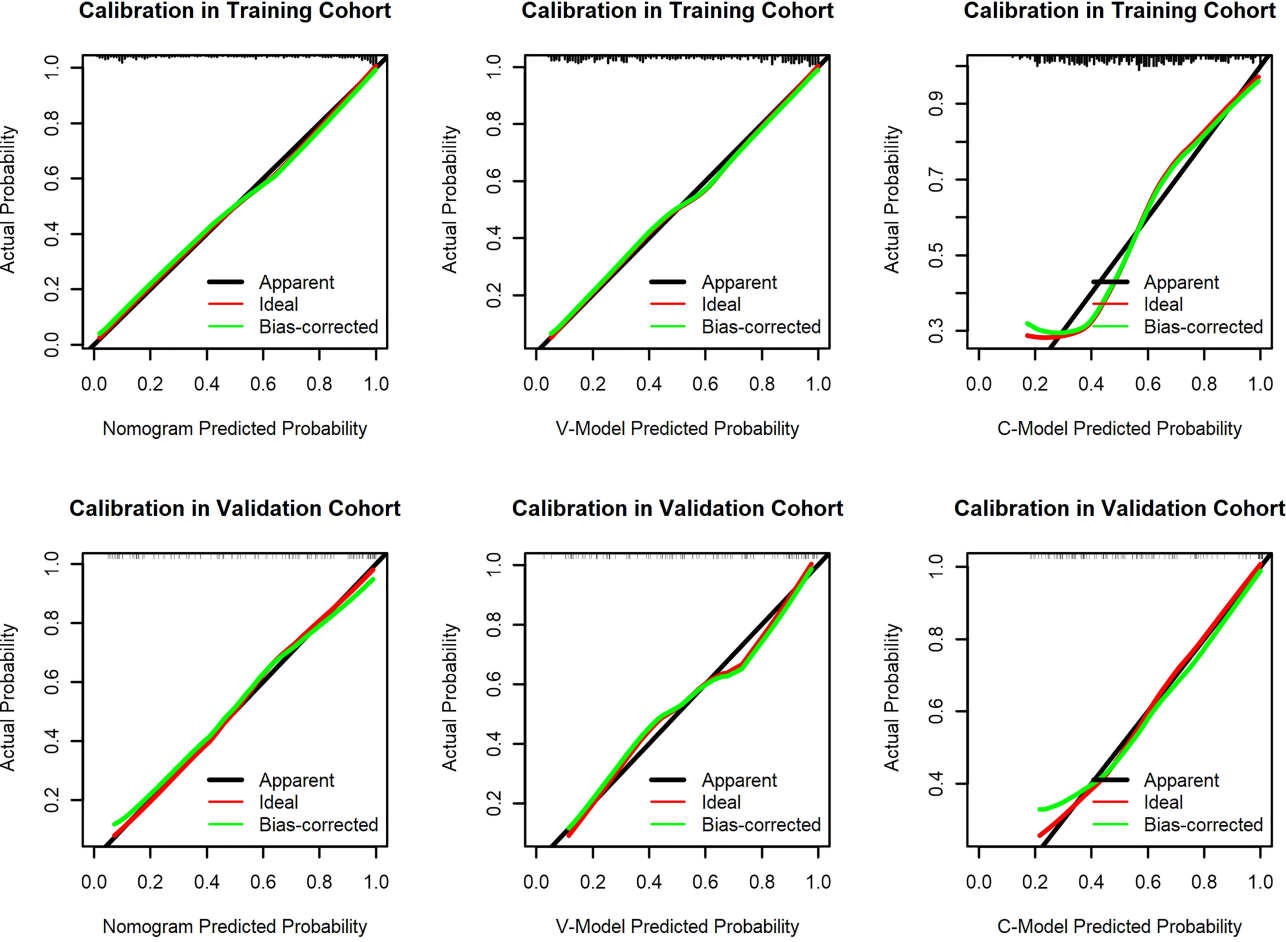
Calibration curves of the constructed models. The y-axis means the actual diagnosed diabetic retinopathy (DR). The x-axis meant the predicted risk of DR. The green lines represent the performance of the training cohort and validation cohort, which indicate a closer fit to the red line. This represents a better prediction.

The calibration curve of the established models evaluated the consistency between actual and predicted probabilities. In this study, it presented remarkable coherence in the three models in both training and validation cohorts (**Figure 5**). However, the P values of the Hosmer–Lemeshow test of the C-Model in both training and validation cohorts (0.263 and 0.374, respectively) were lower than those of the other two models. This illustrated that the V-Model and the nomogram model performed with higher calibration and better fitting.

### Clinical use of the prediction nomogram

**Figure 6:**
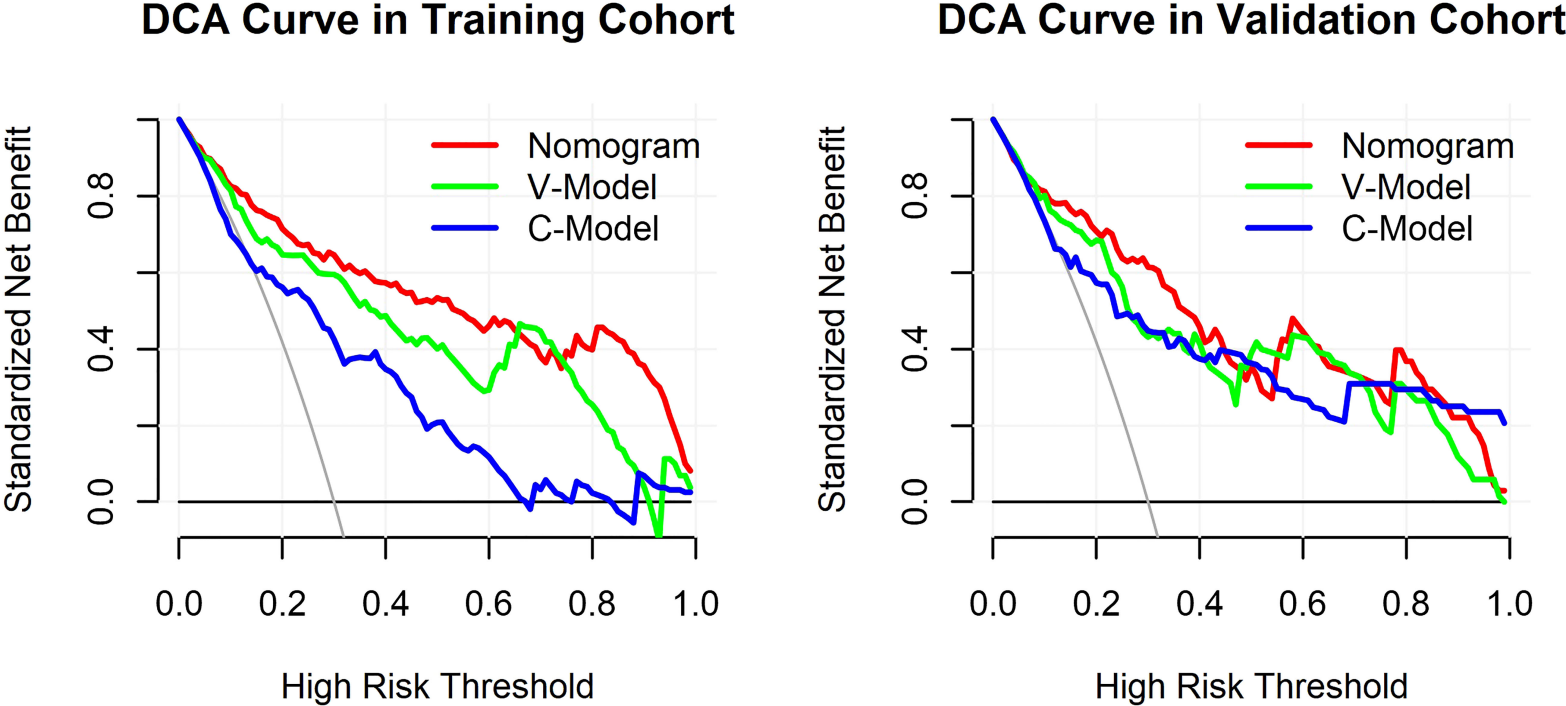
Decision curve analysis (DCA) for the constructed models. The y-axis measured the standardized net benefit. The horizontal line represented that none of the patients has diabetic retinopathy (DR) and all of them were treatment-free. The other oblique line is drawn on the assumption that all of the type 2 diabetes mellitus (T2DM) patients have DR and received clinical intervention.

The results of the DCA for the models are presented in **Figure 6**. The DCA curves were drawn on the basis that the retinopathy prevalence of newly diagnosed diabetes was 30.6% in China (15). The horizontal line represented that none of the patients was living with DR, and all of them were treatment-free. The other oblique line was drawn on the assumption that all of the patients with T2DM had DR and received clinical intervention. The clinical net benefit was calculated using the following formula: net benefit = (true positive + false negative) – (false positive + true negative). For the C-Model, only if the threshold probability was larger than 13% in the validation cohort and in a relatively narrow range in the training cohort, the net benefit of patients was higher than that of the other two extreme curves (horizontal and oblique lines). However, according to the DCA curves, the net benefit of the nomogram and the V-Model was non-zero and much higher than that of the C-Model in a wide range of threshold probability. Moreover, among the established models, the nomogram model gained the highest net benefit in a wide range of threshold probability in both training and validation cohorts. This distinct difference indicated the performance advantage of this nomogram with good clinical safety. Furthermore, the cutoff value (18.9%) of the nomogram model according to the ROC curve of the training cohort was within the range of threshold probability in both DCA curves, showing good clinical usefulness as well. Given that 18.9% is defined as the threshold probability for screening DR and taking medical intervention, the net clinical benefit in the training and validation cohorts was 73.82% and 72.19%, respectively. That is, 73 and 72 of every 100 patients with T2DM who were found to have DR using this nomogram model in the training and validation cohorts would gain clinical benefits, respectively.

## Visualization application of diabetic retinopathy

In a patient with T2DM, the relevant information of risk factors was obtained as follows: fractal dimension, 1.53; venular caliber, 65 μm; arterial tortuosity, 0.0007; insulin dosage, 30 IU/day; and duration of DM, 10 years. According to the predictive nomogram (**Figure 3**), the risk of DR for this patient was approximately 30%, much higher than the cutoff value (18.9%). According to the DCA curve, the medical intervention was supposed to be taken to reduce the risk of incident DR. Meanwhile, a web page was built for screening based on this nomogram model (http://116.205.175.141:4949).

## Discussion

Currently, the nomogram has drawn increasing attention as a predictive tool in diagnosis and prognosis because it can quantify the individual probability of a clinical event with several significant variables (10). It meets our need for early detection with a mathematical model and facilitates individualized medicine (16). Furthermore, the scoring system presents the probability of a clinical outcome clearly and intuitively, promoting the doctor–patient communication and better clinical decision-making (17). In this study, we developed a nomogram using retinal vascular geometry parameters and basic clinical information with no blood test required. Therefore, it may play a key role in screening DR because of its convenience.

A total of 55.61% of the enrolled patients with T2DM had DR. This prediction nomogram model indicated that a smaller fractal dimension, larger venular caliber, increased arterial tortuosity, longer duration of DM, and larger insulin dosage were the key risk factors determining the incident DR for T2DM. Although 14 clinical indicators showed significant differences (P< 0.05) between the DR and NDR groups, only two variables were selected by the LASSO method. The circulation information gained from biological samples, such as blood and urine, was affected by multiple potential comorbidities and personal lifestyles. Whether the accessible circulation information reliably reflected the retinal status and local concentration of any specific factor was worth considering. To assess disease status, it would be optimal to measure indicators directly in the tissues influenced by the disease of interest (18). However, the biopsy of retinal tissue *in vivo* is unrealistic. The evaluation of altered retinal vascular morphology can be a viable alternative. Furthermore, the C-Model showed limited prediction accuracy, while the V-Model displayed favorable predictive performance. However, the predictive value of clinical data could not be ignored as well. In this study, the predictive nomogram developed with retinal vascular factors and clinical factors presented satisfactory predictive capability and better performance than those of the V-Model and C-Model.

The individual risk probability of DR could be obtained by introducing specific indicators of patients into this nomogram. The relatively good discrimination and calibration suggested that this prediction nomogram model could be adapted clinically. Additionally, it would help in risk stratification, leading to better management of patients with and without DR. For individuals at high risk, a closer follow-up was required. This study developed a relatively reliable predictive tool to evaluate the risk of DR in patients with T2DM. Yet, more efforts should be taken to improve the model in terms of accuracy and applicability.

### Duration of disease and insulin dosage

The duration of DM and insulin dosage were risk factors for T2DM. The result implied that longer duration and larger insulin dosages had a close association with incident DR. They should be considered as a pair of risk variables in analyzing the association with DR. Insulin is indispensable for normal protein, fat, and carbohydrate metabolism. Patients with T2DM are initially treated with antiglycemic drugs such as biguanide. However, over time, most of them show a decrease in insulin production; hence, insulin supplementation is required to control chronic high blood sugar levels. Abundant resources greatly satisfy the needs of human beings and influence people’s behavior and lifestyle. An unhealthy lifestyle is the main reason for the shift in the therapeutic regimen for patients with T2DM (19), starting with glucose-lowering medicines, followed by low-dose insulin, and then increasing the insulin dosage. Over time, the function of islet cells is impaired with insufficient insulin secretion, leading to a growing demand for exogenous insulin dosage (20).

In our study, the mean duration of the disease course was approximately 6 years in NDR and 9 years in DR in both training and validation cohorts. The use of mean insulin dosage in DR was much more than that in NDR in both cohorts. Thus, we could infer that the insulin dosage was roughly parallel with the duration of T2DM. Long-term hyperglycemia can damage the vascular system insidiously, including the retinal vascular tree. Studies also showed that a longer duration of DM led to a higher prevalence of DR (21, 22). Based on this perception, the duration of DM may be a more vital factor to the risk of retinopathy. Meanwhile, the regression coefficient of the duration of DM was larger than that of insulin dosage, suggesting that the duration was a more important one contributing to incident DR. The patients with a longer duration of T2DM and a larger dosage of insulin were more likely to suffer from DR.

### Retinal geometry

Previous researchers reported an optimal circulation that drove blood flow with the least energy and completely met the metabolic demands of local tissues (23). Any deviation from this optimal vascular structure would result in reduced circulatory efficiency. The primary functions of the retinal vascular tree were nourishing the retinal tissue and removing local wastes. As a result, the retinal vascular needed to modify itself continuously to accommodate the changing demand for metabolism and blood pressure. Therefore, the peripheral metabolic environment and perfusion demands were of paramount importance to determine the ultimate retinal vascular architecture, especially in metabolic diseases, such as diabetes.

### Vascular tortuosity

The normal retinal vascular is gently curved or roughly straight. A tortuous vascular may result from increasing vascular endothelial growth factor (VEGF) levels in response to an incomplete retinal vessel wall caused by hypoxia or ischemia (24). Except traditional neovascularization, tortuosity is considered as another form of angiogenesis with an increased VEGF level (25) because it must elongate enough to accommodate its tortuous changes. This appears to be consistent with the conventional view of the retinopathy signs of venular beading. Yet, in this study, both arterial and venular vascular showed a statistically significant difference between NDR and DR in terms of tortuosity. The coefficient of venular tortuosity shrunk to zero in the LASSO analysis, implying a less predictive value in this study than that of arterial tortuosity.

The hyperglycemia-mediated retinal vascular injury started with the loss of endothelial cells and surrounding pericytes, resulting in autoregulation dysfunction (26). The chronic high blood sugar level undermined the basement membrane of the vessel wall. The collagen fibers of the basement membrane functioned against the irregular longitudinal traction and transmural pressure with its axial strain, maintaining the morphological stability of the retinal vasculature (27). The increased vascular tortuosity indicated a decrease in endothelial cells and pericytes (28), thus being relatively more fragile and vulnerable to hemorrhage. This was parallel with well-recognized features of retinopathy in diabetes. We inferred that patients with tortuous retinal vessels suffered from more severe retinopathy and were more likely to be observed with hemorrhage spots under a funduscope. Moreover, neovascularization might appear much earlier than less tortuous retinal vasculature.

### Vascular caliber

The biological mechanism of the changes in the retinal vessel caliber in patients with DR is unclear. The widening venule may reflect increased volumetric blood flow related to hyperglycemia and retinal hypoxia. The uncontrollable blood sugar and tissue hypoxia would finally result in accumulative lactic acidosis and autoregulatory endothelial dysfunction (29). The retinal vascular caliber largely depends on retinal perfusion and vascular autoregulation. Blunt vasomotion caused by impaired endothelial cells may play a role in this. Furthermore, retinal perfusion is regulated by the demands of local metabolism. A smaller vascular caliber has been reported in patients undergoing panretinal photocoagulation (30). The reduced demand of the metabolism secondary to the damage of the ischemic retinal area was assumed to be responsible for narrowed retinal blood vessel with improved oxygenation and lower volumetric blood flow. Furthermore, a close connection was reported between the gradually increased venular caliber and higher HbA1c level (31), as well as the severity of retinopathy (32), which reflected poor blood glucose control indirectly.

In this study, a significant difference in the venular caliber was observed between DR and NDR, while no obvious difference was found in the arterial caliber. The observed result was consistent with the findings of population-based studies (32, 33). In elderly patients without DM, both arteriolar and venular calibers decreased with age, and the narrowing retinal vascular was proven to be associated with hypertension and cardiovascular risk (34, 35). It was assumed that the adverse effect of hyperglycemia was underestimated, and poor control of blood sugar level might exert a greater influence on vascular architecture than blood pressure (36, 37).

### Fractal dimension

The fractal dimension is a relatively simple method used for evaluating the complexity of the retinal vascular tree. It is not associated with macrovascular disease and has a closer association with microvascular disease (38). As DR progresses, further non-perfusion patches formed with occlusive microvasculature and the complexity of retinal vascular pattern reduces with a decrease in fractal dimension (39, 40).

Generally, the retinal vascular fractal dimension decreases in vessel-involving diseases, including DR, which is in agreement with the findings of our study. However, increased fractal dimension in DR was also reported in previous studies. The disorganized morphology of neovascularization may be responsible for this. In this study, among the five selected variables, fractal dimension had the largest absolute value of the coefficient with a P value<0.0001, indicating that the fractal dimension had the greatest impact on the predictive probability. This was parallel with the point that fractal analysis was a potential predictive factor for diagnosing DR (41).

Currently, the preventive measures for retinopathy in T2DM are limited to blood sugar control and a healthy lifestyle. Limited therapeutic measures can slow down the progression of DR but can never cure it completely. In clinical practice, identifying patients with DR early and precisely determining the timing for intervention are worth considering. Meanwhile, the retinal vascular geometry is closely related to tissue metabolism, reflecting retina status directly. This study developed and validated a prediction nomogram model based on retinal vascular indicators for retinopathy in T2DM and assessed its clinical usefulness. In our study, the DCA curve suggested that the observed net clinical benefit of the nomogram was much higher than that of reference lines in a wide range of threshold probability. Considering the cutoff value (18.9%) as the threshold probability of the DCA curve, no clinical interventions should be taken for patients with probabilities lower than 18.9%. However, if the probability of DR is more than 18.9%, further examination can be arranged and a close follow-up is required. Based on this perception, this prediction model facilitates risk stratification and clinical decision-making in patients with T2DM.

## Conclusion

In conclusion, the five identified independent risk variables for DR in T2DM in our study were fractal dimension, venular caliber, arterial tortuosity, duration of DM, and insulin dosage. Additionally, we developed a personalized nomogram model and a further web page, making it more convenient for clinical application. This predictive nomogram presented a wide range of probability thresholds, indicating good clinical application. This can facilitate early detection and risk stratification of developing DR.

### Limitations

This study still had some limitations. First, the enrolled population was relatively small. All of the recruited participants were hospitalized patients mainly distributed in surrounding communities. No biochemical factors were introduced in this nomogram model. Further studies are supposed to be conducted to confirm the contribution of biochemical risk factors in the diagnosis of DR. Therefore, extending the range of participants might make the nomogram model more representative. Also, the duration of DM is an arbitrary variable because most patients were not aware of it when they were initially in such a state. Furthermore, no patient could remember the exact date even the month of diagnosis precisely. Third, the venular diameter was the only variable influenced by magnification in this model. These magnification differences caused by refractive error were unavoidable among individuals, leading to slight differences in the measurement of vascular diameter. Therefore, prospective studies should be implemented further to confirm the diagnostic value of the retinal vascular caliber.

## Data availability statement

The raw data supporting the conclusions of this article will be made available by the authors, without undue reservation.

## Ethics statement

The studies involving human participants were reviewed and approved by Medical Ethics Committee of the Second Affiliated Hospital of Chongqing Medical University. The patients/participants provided their written informed consent to participate in this study.

## Author contributions

Study conception and design: MW and XZ; retinal traits measurement: SL and ZZ; data collection and data analysis: MW and XZ; original manuscript writing: MW; manuscript revising: XZ, DL, and ZZ; web page design and construction: JC. All authors contributed to the article and approved the final manuscript.

## Funding

This study was supported by National Natural Science Foundation of China (No.82070976) and Future Medical Youth Innovation Team Development Support Program of Chongqing Medical University.

## Acknowledgments

We thank all the patients who allowed their data to be used for this study. We gratefully thank the doctors and nurses of orthopedics and internal medicine at Second Affiliated Hospital of Chongqing Medical University for the help in this study.

## Conflict of interest

Author SL is employed by EVision technology Beijing co.LTD, China.

The remaining authors declare that the research was conducted in the absence of any commercial or financial relationships that could be construed as a potential conflict of interest.

## Publisher’s note

All claims expressed in this article are solely those of the authors and do not necessarily represent those of their affiliated organizations, or those of the publisher, the editors and the reviewers. Any product that may be evaluated in this article, or claim that may be made by its manufacturer, is not guaranteed or endorsed by the publisher.
